# Hand, Foot, and Mouth Disease in Adults

**DOI:** 10.7759/cureus.48387

**Published:** 2023-11-06

**Authors:** Catarina Afonso, Ana Almeida

**Affiliations:** 1 Family Medicine, Unidade de Saúde Familiar (USF) Tornada, Caldas da Rainha, PRT

**Keywords:** viral illness, coxsackievirus, enterovirus, exanthem, hand-foot-mouth disease

## Abstract

Hand, foot, and mouth disease (HFMD) is a viral infection primarily affecting children, but it can occur in individuals of all ages. In this article, we present the atypical case of a 36-year-old male with no significant medical history who recurred to the emergency department with a three-day history of fever, sore throat, and a maculopapular rash. After an extended evaluation with normal blood tests, he was discharged home with a diagnosis of HFMD. This case underscores the importance of considering HFMD when evaluating adult patients with rashes, and patients should be reassured about the self-limiting nature of the disease.

## Introduction

Hand, foot, and mouth disease (HFMD) is a well-known viral infection that predominantly affects children. It is typically characterized by a triad of symptoms, including fever, sore throat, and the development of painful and characteristic lesions on the hands, feet, and oral mucosa [1,2]. While HFMD is considered a common childhood illness, there have been increasing reports of its occurrence in adults [3]. This case report aims to shed light on the presentation of HFMD in an adult patient, emphasizing the importance of recognizing this condition beyond the pediatric population.

Although HFMD is primarily caused by Coxsackie virus or enterovirus and is considered a mild and self-limiting illness in children, its occurrence in adults can present unique challenges in diagnosis and management. The clinical course and symptoms of HFMD in adults can be more severe and may mimic other dermatological conditions, leading to potential misdiagnoses and delays in appropriate care [2].

In this case report, we present the clinical history, examination findings, laboratory results, and management of a 36-year-old adult male who presented with HFMD. This case underscores the significance of considering HFMD in the differential diagnosis of adults presenting with febrile exanthems and oral symptoms, particularly in the absence of common risk factors such as recent travel or exposure to infected children. Understanding the atypical presentation of HFMD in adults is crucial for early diagnosis, appropriate management, and preventing potential complications.

## Case presentation

This article presents the case of a 36-year-old male with no significant medical history who presented to the emergency department with a primary complaint of a rash associated with fever and a painful throat.

The patient reported a three-day history of an evolving rash, initially characterized by erythematous patches that later progressed into a maculopapular rash localized around the mouth, palms of the hands, and soles of the feet. Alongside the rash, he complained of fever and odynophagia, which prompted his hospital visit. The patient had an up-to-date Portuguese national vaccination record and denied recent travel or close contact with individuals exhibiting similar symptoms.

Upon physical examination, he exhibited a febrile state and maculopapular lesions primarily distributed around his perioral region, which extended to involve the palms of his hands and the soles of his feet. He had a fever between 37.9ºC and 39ºC, which responded to paracetamol. In response to the patient's symptoms, a comprehensive laboratory workup was performed, including a complete blood count, C-reactive protein (CRP), liver function tests, and a venereal disease research laboratory (VDRL) test. All laboratory results fell within normal limits, with no significant abnormalities detected.

Based on the distinct clinical presentation and the typical progression of symptoms, the patient received a diagnosis of HFMD. To manage his symptoms, he was prescribed a therapeutic regimen comprising paracetamol, ibuprofen, and cetirizine. Following the confirmation of the diagnosis, the patient was discharged from the hospital with instructions for symptomatic relief and self-care at home.

However, despite the initial treatment and discharge, the symptoms persisted, prompting the patient to seek follow-up care from his family physician. During the follow-up visit, one day after the ER episode, he reported that the lesions had initially appeared in his oral mucosa and perioral region, subsequently spreading to involve the palms of his hands and the soles of his feet. Additionally, he continued to experience a sore throat and fever.

**Figure 1:**
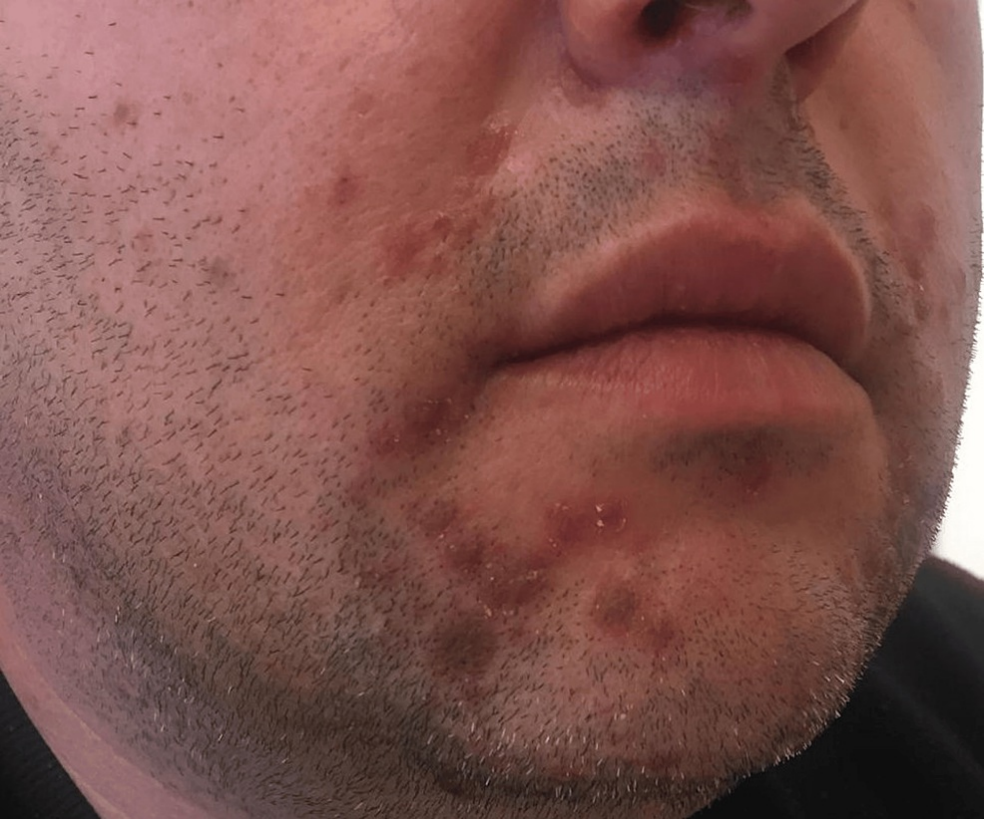
Perioral lesions.

**Figure 2:**
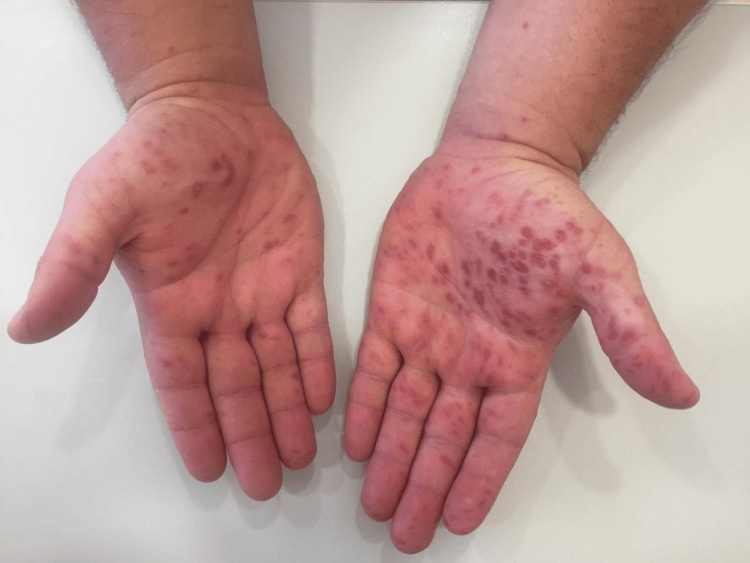
Hand lesions.

During the follow-up appointment, characteristic HFMD lesions were still visible on the perioral region, hands, and feet, consistent with the initial diagnosis (Figures 1 and 2).

To address the ongoing pain and discomfort, the management plan was optimized, and the patient received additional instructions for at-home care, with a focus on pain management and comfort. It was also explained that it is important to isolate from others, especially young children, to prevent transmission. The patient was advised to do frequent hand washing with soap and water, stay hydrated, and avoid acidic or spicy foods to alleviate mouth sore. Adequate rest and sleep support the immune system, while cleaning and disinfecting personal items can prevent the virus from spreading. Furthermore, the patient had a full recovery from this episode two to three weeks later.

## Discussion

The presented case highlights an atypical occurrence of HFMD in an adult, a condition primarily associated with children [4]. HFMD is typically caused by Coxsackie virus or enterovirus, leading to characteristic symptoms of fever, sore throat, and the development of painful vesicular lesions on the hands, feet, and in the oral cavity [5,6].

One of the primary aspects of this case that deserves attention is the age of the patient. While HFMD is a common childhood illness, adult cases are relatively rare [7]. The patient's initial presentation of a maculopapular rash, painful throat, and fever mimicked several other viral infections. It is important for healthcare providers to recognize that HFMD can affect adults and should be considered in the differential diagnosis of adult patients with similar symptoms, particularly in the absence of a recent travel history or known exposure to infected children [6-8].

The presentation of HFMD in this adult patient was atypical, as the initial lesions were observed in the oral mucosa and perioral region before extending to involve the palms of the hands and soles of the feet. This pattern contrasts with the typical progression seen in children, where the extremities are usually affected first [5]. The painful throat, or odynophagia, is a common symptom of HFMD, yet its presence in an adult patient may not immediately raise suspicion of this childhood-associated disease.

While individual cases of HFMD can occur sporadically as in this case, the disease often gains attention when it appears in outbreaks or clusters mostly related to children [9]. This case serves as a reminder of the importance of patient education and public health awareness [1]. HFMD is a highly contagious disease, and adults who contract it can serve as sources of transmission. Promoting awareness and preventive measures, such as good hand hygiene and isolation of affected individuals, is critical to curb the spread of HFMD within communities.

Preventing the transmission of HFMD involves several key measures. Good hygiene practices, such as frequent hand washing with soap and water, can help reduce the risk of infection. Avoiding close contact with individuals who have HFMD, especially those displaying symptoms, is important in preventing transmission. Disinfecting commonly touched surfaces and toys can also be beneficial. Individuals diagnosed with HFMD should isolate themselves to minimize contact with others, and caregivers should ensure strict adherence to these precautions, particularly in childcare and school settings, where outbreaks are common. Education and awareness campaigns on proper hygiene and the importance of early isolation can further contribute to reducing the spread of the virus and controlling HFMD outbreaks.

Through this case report, we aim to contribute to the growing body of evidence regarding HFMD in adults, fostering a better understanding of the disease's clinical spectrum and highlighting the importance of timely recognition and management in adult patients.

## Conclusions

In conclusion, this case report highlights the necessity for healthcare providers to consider HFMD as a potential diagnosis in adults with unexplained febrile exanthems and oral symptoms, especially when typical risk factors are absent. Atypical presentations, though rare, do occur, and recognizing them is essential for accurate diagnosis and appropriate patient management. Additionally, public health campaigns should continue to stress the importance of preventive measures to mitigate the transmission of HFMD, even in cases involving adults. Further research and case reports can contribute to a better understanding of HFMD in adults, enhancing early recognition and treatment.
